# Rapid and sustained effect of anti-TNF treatment in patients with ADA2 deficiency

**DOI:** 10.1186/1546-0096-13-S1-O80

**Published:** 2015-09-28

**Authors:** R Caorsi, A Omenetti, A Morreale, A Insalaco, A Buoncompagni, P Picco, C Malattia, C Gandolfo, I Aksentievic, A Martini, M Gattorno

**Affiliations:** 1 G. Gaslini Institute, 2nd Division of Pediatrics, Genova, Italy; 2 University of Genova, Department of Pediatrics, Genova, Italy; 3 Ospedale Pediatrico Bambino Gesù, Department of Pediatrics, Roma, Italy; 4 G. Gaslini Institute, Department of Radiology, Genova, Italy; 5 National Human Genome Research Institute, Bethesda, MD, USA

## Introduction

Mutations of CERC1 have been recently reported as causative of an inflammatory condition characterized by polyarteritis, cerebral stroke and immunodeficiency; the response to immunosuppressors and biological drugs is not univocal.

## Aim of the study

To describe a series of patients with DADA2, focusing on the response to treatment and outcome.

## Patients and methods

Patients with a clinical history consistent with a possible DADA2 were retrospectively analyzed; molecular analysis of the CECR1 gene was performed. Detailed analysis of the clinical presentation, disease course and response to treatment was retrospectively performed in patients with confirmed diagnosis.

## Results

The retrospective analysis of patients allowed to identify 5 patients presenting with a strong clinical suspicion of DADA2. The mean age of disease onset was 12 months (range 6-36). The disease course was chronic in two patients and recurrent in three. All patients presented skin involvement and elevation of acute phase reactants; three patients presented multiple strokes, one patient acute invagination of the small bowel. Skin biopsy was consistent with PAN in three patients.

The molecular analysis of CECR1 gene identified homozygosis or compound heterozygosis for deleterious mutations (G47R, G47A, P251L, R312X, E328D, T360A) in all patients.

All patients required high doses of steroids to control the skin manifestations and the systemic inflammatory features but a clinical relapse was observed at the time of steroid tapering. In two patients thalidomide was able to completely control the disease manifestations while one patient presented a partial response. Other immunosuppressant (oral cyclophosphamide and cyclosporine) were not able to control the disease activity; treatment with anakinra was tempted in one patient, without clinical improvement. After the suspension of thalidomide and the failure of cyclophosphamide, etanercept was started in one patient on may 2008 at the dose of 0.8 mg/kg/daily. The patients presented a rapid and complete control of the skin manifestations with a rapid normalization of acute phase reactants, despite the withdrawal of steroidal treatment. The same brilliant response to etanercept was also observed in the brother and, more recently, in other three patients. The median duration of treatment with anti-TNF agents is now 34 months (range 6 months-7 years). All 5 patients display a complete control of clinical manifestations and laboratory parameters and are off from any steroid treatment. No severe infectious or other complications have been described so far.

## Conclusion

This series of 5 patients with DADA2 enlightens the long-term efficacy of anti-TNF agents.

